# Population growth of Mexican free-tailed bats (*Tadarida brasiliensis mexicana*) predates human agricultural activity

**DOI:** 10.1186/1471-2148-11-88

**Published:** 2011-04-01

**Authors:** Amy L Russell, Murray P Cox, Veronica A Brown, Gary F McCracken

**Affiliations:** 1 Department of Biology, Grand Valley State University, Allendale, MI 49401, USA; 2 Institute of Molecular BioSciences, Massey University, Palmerston North 4442, New Zealand; 3 Allan Wilson Centre for Molecular Ecology and Evolution, Palmerston North, New Zealand; 4 Bio-Protection Research Centre, Canterbury, New Zealand; 5 Department of Ecology and Evolutionary Biology, University of Tennessee, Knoxville, TN 37996, USA

## Abstract

**Background:**

Human activities, such as agriculture, hunting, and habitat modification, exert a significant effect on native species. Although many species have suffered population declines, increased population fragmentation, or even extinction in connection with these human impacts, others seem to have benefitted from human modification of their habitat. Here we examine whether population growth in an insectivorous bat (*Tadarida brasiliensis mexicana*) can be attributed to the widespread expansion of agriculture in North America following European settlement. Colonies of *T. b. mexicana *are extremely large (~10^6 ^individuals) and, in the modern era, major agricultural insect pests form an important component of their food resource. It is thus hypothesized that the growth of these insectivorous bat populations was coupled to the expansion of agricultural land use in North America over the last few centuries.

**Results:**

We sequenced one haploid and one autosomal locus to determine the rate and time of onset of population growth in *T. b. mexicana*. Using an approximate Maximum Likelihood method, we have determined that *T. b. mexicana *populations began to grow ~220 kya from a relatively small ancestral effective population size before reaching the large effective population size observed today.

**Conclusions:**

Our analyses reject the hypothesis that *T. b. mexicana *populations grew in connection with the expansion of human agriculture in North America, and instead suggest that this growth commenced long before the arrival of humans. As *T. brasiliensis *is a subtropical species, we hypothesize that the observed signals of population growth may instead reflect range expansions of ancestral bat populations from southern glacial refugia during the tail end of the Pleistocene.

## Background

Modern human populations and their activities have had a significant, and frequently negative, impact on other organisms [1-3]. Human populations exert a tremendous ecological and evolutionary pressure on native species, comparable in total effect to that of glaciations on temperate species but operating over much shorter timescales (10^1^-10^3 ^versus 10^4^-10^5 ^years; [4,5]). The effect of human activities on native species is not easily predictable, and in some instances human activities have benefited native wildlife, often through increasing habitat or food availability [6,7]. Genetic methods are starting to prove useful for linking demographic processes in animal species to human activity, with recent studies attributing decreasing effective population size and increasing population fragmentation to anthropogenic deforestation, the expansion of agriculture, road construction and the presence of human settlements [8-10]. Here, we use genetic analyses to investigate whether anthropogenic forces, specifically the spread of agriculture, or non-anthropogenic forces such as the retreat of the Laurentide and Cordilleran glaciers, have driven the population expansion of a North American bat.

Roosting colonies of Mexican free-tailed bats (*Tadarida brasiliensis mexicana*) are some of the largest and most conspicuous aggregations of bats in North America. While historic colony counts numbered in the tens of millions [11,12], more recent, and likely more accurate, exit counts using thermal imaging and infrared tracking methods estimate the total census size of free-tailed bat colonies throughout the entire southwestern United States at 9 million individuals [13]. However, when the southwestern United States and Mexico are considered together, the census population size of this species may easily reach 10^7^-10^8 ^individuals, making it one of the most numerous known non-human mammals. The largest known aggregations of Mexican free-tailed bats are in nursery colonies, primarily hosting reproductive adult females and their young. During the energetically demanding period of pregnancy and lactation, females ingest up to two-thirds of their body weight in insects every night [14]. These colonies thus depend upon a large and reliable base of insect prey to maintain their considerable population sizes.

A number of studies have documented strong links between Mexican free-tailed bats and important agricultural pest insects, especially *Helicoverpa zea *and *Spodoptera exigua *(Lepidoptera: Noctuidae). The adults of these moth species migrate northwards in the spring from Mexico to the United States, often flying at high altitudes to take advantage of prevailing winds [15]. High-altitude echolocation surveys show that *Tadarida *feeding calls are coincident in time and altitude with these migrating insect populations [16]. Molecular analyses also document significant levels of *H. zea *and *S. exigua *DNA in the feces of Mexican free-tailed bats [17], thus further verifying this predator-prey relationship. Although the diet of Mexican free-tailed bats is not restricted to agricultural pest insects [18], the development of human agriculture likely resulted in predictable swarms of pest insects caused in part by increasing levels of plant cultivation. Mexican free-tailed bats now exploit this resource heavily, especially during pregnancy and lactation [19].

This predator-prey relationship between Mexican free-tailed bats and agricultural pest insects suggests that we should observe population growth in bats coupled to increases in insect prey in connection with human agricultural practices in the Americas. Population growth would most likely be associated with the widespread expansion of European agriculture during the last few centuries, or as an outside possibility, with the emergence of Native American agriculture during the last few thousand years [20]. More concretely, population growth of Mexican free-tailed bats cannot be linked to anthropogenic processes if it occurred before humans arrived in the Americas around 9-15 thousand years ago (kya) [21].

Population growth leaves distinct patterns of variation in genetic data, and these patterns can be used to infer effective sizes and growth rates, as well as estimate the time at which growth commenced [22]. Genetic variation in *T. b. mexicana *is consistent with population growth; mismatch distribution analyses of mitochondrial DNA sequence data are suggestive of population expansion occurring in concert with the development of human agriculture (2.7-3.0 kya; [23]). However, these analyses lack statistical power to distinguish between anthropogenic and climatic drivers of population growth, and this particular test often exhibits high false positive rates [24]. In this paper we take advantage of more statistically rigorous methods to infer these demographic parameters and evaluate the anthropogenic influence hypothesis. Here, we explore the rate and time of population growth in Mexican free-tailed bats using one of these advanced inferential methods, approximate Maximum Likelihood. A quantitative analysis of the rate and timing of population growth in *T. b. mexicana *can help us understand the response of wildlife species to major innovations in human cultural evolution such as the development of agriculture.

## Results

### Approximate maximum likelihood

Summary statistics suggest that Mexican free-tailed bat populations have experienced growth (Table 1), as do published neutrality tests and mismatch distributions [23]. Genetic diversity is particularly high for the mtDNA locus (*θ_W _*= 0.068 per bp), whereas diversity for the *RAG2 *gene is an order of magnitude lower (*θ_W _*= 0.0065 per bp). However, this disparity primarily reflects differences in the mutation rates of these two loci (i.e., 2.0 × 10^-8 ^and 8.6 × 10^-10^/bp/year, respectively). More tellingly, singleton polymorphisms - a signal of population growth - are frequent in both datasets, accounting for 40% of all polymorphisms in the mtDNA control region and 32% of all polymorphisms in the *RAG2 *locus. Reflecting these high levels of singletons, Tajima's *D *is negative for both loci (*TD *= -1.5 for the control region and *TD *= -0.82 for *RAG2*). Although such values are broadly indicative of growth, more sophisticated analyses are necessary to statistically exclude the possibility that Mexican free-tailed bat populations have actually remained constant in size and, if such a model can be rejected, to infer the rate and time of onset of their growth.

**Table 1:** Summary statistic information for the haploid mtDNA control region and the autosomal *RAG2 *locus

Summary Statistic	Symbol	Control region (mtDNA)	*RAG2* (autosomal)
Sample size (chromosomes)		94	150
Sequence length (bp)		474	686
Segregating sites	S	154	25
Watterson's theta (per bp)	θ_W_	0.068	0.0065
Pairwise differences (per bp)	θ_π_	0.038	0.0047
Tajima's *D*	TD	-1.5	-0.82
Singletons	η_1_	62	8
Haplotypes	h	86	52

**Figure 1:**
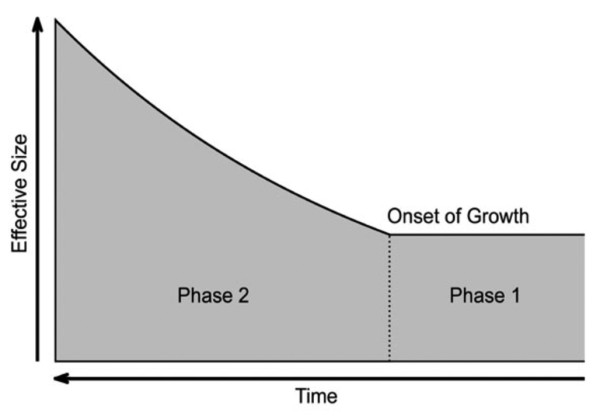
**Two-phase model of population growth**. An early period of constant size (Phase 1) is followed by a period of population growth (Phase 2). The dotted line reflects the time of onset of growth, during which the effective population size increases exponentially from ancestral to modern levels.

**Figure 2:**
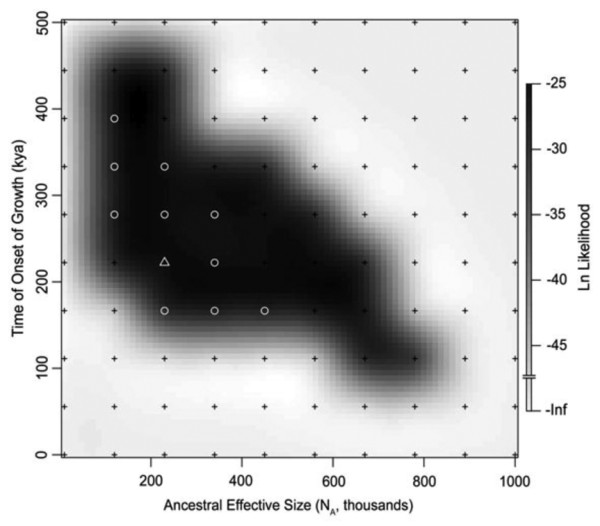
**Combined log-likelihood surface (*N_A _*versus *τ*) for the haploid mtDNA control region and autosomal *RAG2 *locus**. Black and white points indicate the grid of sampling locations. Log-likelihoods at these points are known with certainty, whereas log-likelihoods in the intervening spaces are interpolated. Regions of the parameter space with highest likelihood are shaded black. Only highlighted white points (circles and triangles) fall within the 95% confidence interval. The maximum likelihood estimate (MLE) is indicated by a white triangle. *N_0 _*is set to its value for the MLE.

We turned to approximate Maximum Likelihood to explore a two-phase population growth model (Figure 1). We applied this method to our empirical dataset, first, to the two loci individually, and subsequently, to the two loci together (Table 2). Considering the mtDNA control region alone, the Maximum Likelihood estimate (MLE) suggests an ancestral effective population size of ~120 thousand, a modern effective population size of ~11 million, and an onset of growth of ~330 kya (Additional file 1). Considering *RAG2 *alone, the MLE suggests an ancestral effective population size of ~340 thousand, a modern effective population size of ~6 million, and an onset of growth of ~110 kya (Additional file 2). Importantly, however, the actual demographic history that produced the observed data must be compatible with both loci taken together; thus, to determine the demography that best fits both of our genetic loci, we considered the combined likelihood for the mtDNA control region and autosomal *RAG2 *locus (Figure 2). Our combined MLE favors an ancestral effective population size of ~230 thousand, a modern effective population size of ~12 million, and an onset of growth ~220 ka. Only 63 grid points (of 1000, i.e., 6.3%) within the 3-dimensional parameter space of *N_A_*, *N_0_*, and τ are significantly likely (i.e., these points form the 95% confidence interval) (Additional file 3, points ranked by likelihood). This set includes ranges of ~120-450 thousand for *N_A_*, ~6-50 million for *N_0_*, and ~120-500 kya for *τ*. We note that the point representing constant effective size (i.e., no growth, *N_0 _*= *N_A_*, or equivalently, *α *= 0) is rejected with strong statistical significance. In general, the ancestral effective size *N_A _*and the time of onset of growth *τ *were inferred with some certainty. The haploid locus contains more information about time (cf. Additional file 1), whereas the autosomal locus, with its larger effective size and thus older time to the most recent common ancestor (TMRCA), carries more information about the ancestral effective size (Additional file 2). We had less power to infer the modern effective size *N_0_*, as is evident from the profile likelihood curve derived from the combined likelihood surface (i.e., generated for *N_A_*, *N_0 _*and *τ *separately; Additional file 4). However, this uncertainty in *N_0 _*is accommodated naturally by the likelihood function, and is thus accounted for in all the demographic values that we present here.

**Table 2:** Maximum likelihood estimates for modern and ancestral effective population sizes, time of onset of growth and population growth rates

Demographic Parameter	mtDNA		*RAG2*		Combined	
	
	MLE	95% CI	MLE	95% CI	MLE	95% CI
N_A _(×10^3^)	120	10-890	340	120-560	230	120-450
N_0 _(×10^6^)	11	6-50	6	6-50	11	6-50
τ (kya)	330	110-500	110	0-500	220	110-500
α (×10^-5^/generation)	5.4	2.5-18	10	2.6->>100	7	2.6-18
Fold growth	93	10-3900	16	10-420	48	16-320

Note too that our three demographic parameters (i.e., *N_A_*, *N_0_*, and *τ*) are not independent, but are instead correlated to various extents. Considering grid points within the 95% confidence interval of our combined likelihood surface (Additional file 3), the two effective sizes (*N_A _*and *N_0_*) are weakly correlated (Spearman's rank correlation; *r_s _*= 0.32, *P *= 0.01). This explains ~11% of the observed variance. However, the interdependence of the effective population sizes and the time of onset of growth is more pronounced. We find that *τ *is very strongly, negatively correlated with *N_A _*(*r_s _*= -0.68, *P *<< 0.0001) and *N_0 _*(*r_s _*= -0.76, *P *= 0. 0001), thus explaining ~46% and ~58% of the observed variance, respectively. Both associations are non-linear, and in each case, the time of onset of growth appears younger as the effective size increases. This suggests that development of new summary statistics with increased power to distinguish effective population sizes and/or the time of onset of growth would help to reduce the size of our 95% confidence interval by decoupling this parameter dependence.

### Validation

Even though we used only standard methods, we still validated our inference technique using data simulated under known demographic histories. To do so, we generated coalescent trees and ancestral recombination graphs (ARG) at 10^3 ^values of *N_A_*, *N_0_*, and *τ *drawn randomly from a uniform distribution across the parameter space, and calculated the values of *S*, *η_1_*, and *h *returned by each of these simulations. For each dataset, we then applied the approximate Maximum Likelihood algorithm described above, and calculated 95% confidence intervals for *N_A_*, *N_0_*, and *τ*. We considered individual test cases to be successful when our *inferred *demography included the *known *(i.e., defined) values of *N_A_*, *N_0_*, and *τ *within its 95% confidence intervals. By setting the type I error rate at 0.05, we would just by chance expect to infer *N_A_*, *N_0_*, and *τ *incorrectly for ~5% of our simulated datasets. In practice, we observe a somewhat higher type I error rate of 9% (i.e., a marginally enlarged coverage of the confidence interval). We suspect that this small difference, which causes our demographic estimates to appear slightly conservative, probably occurs because we must calculate likelihoods at points along a grid of parameter values, rather than inferring them freely across the entire likelihood surface. Although decreasing the grid spacing (say, to either a 100 × 100 × 100 grid, or the point where we could return the complete likelihood surface) would then systematically reduce our observed type I error rate, this option is not currently computationally feasible. The existing likelihood surface based on a 10 × 10 × 10 grid required approximately three thousand CPU hours to calculate on a fast distributed-computing system. In comparison, a larger 100 × 100 × 100 grid would take an impossible three million CPU hours to compute (equivalent to 343 years running on a single computer). We note, however, that the algorithm applied here varies only very slightly from expectations, and furthermore, because these small errors are conservative, they do not materially affect any of our main conclusions.

## Discussion

We set out to determine whether Mexican free-tailed bat populations have experienced population growth and, if so, whether their onset of growth was concurrent with the expansion of human agricultural activity. The approximate Maximum Likelihood method applied here is a flexible and powerful analytical tool for testing hypotheses about historical demography. Similar methods have been applied previously to demographic analyses of human populations [22,25,26], and have relied on large datasets to infer complex demographic models [27]. Here, we employ these methods to address a specific aspect of the historical demography of Mexican free-tailed bats, namely, the time of onset of population growth. Using coalescent-based inferential statistics, we directly address our hypothesis while ensuring that our data have sufficient power. Further, we address our hypothesis using amounts of sequence data that are typically available for many non-model organisms (only ~1.2 kb). We find that these bat populations have grown, but that their population growth began long before the arrival of humans in the Americas.

Several key points emerge from our analyses. First, a scenario whereby Mexican free-tailed bat populations are not growing (i.e., they are constant sized) is statistically unlikely. Considering the data points contained within the 95% confidence intervals for the demographic parameters (Additional file 3), modern effective sizes were always estimated to be larger than ancestral effective sizes (i.e., *N_0 _*≠ *N_A_*). We therefore have statistical confidence that Mexican free-tailed bat populations have increased in size. Second, growth rates are not particularly large. The MLE of the combined likelihood surface suggests ~48-fold growth from the ancestral to modern effective population size (range: 16- to 324-fold growth). This rate of increase (MLE *α *= 7×10^-5^/generation) is equivalent to a doubling of the bat population approximately every 31 kyr (or ~7,725 generations). Such values do not suggest the extremely rapid growth that would be expected if Mexican free-tailed bat populations expanded from a small population size to their current numbers in response to human agricultural activity during the last few hundred years. Third, although we have little statistical power to place an upper bound on the time of onset of growth, our analysis has considerable power to infer the lower bound (cf. Additional file 4). Our MLE suggests that population growth in Mexican free-tailed bats began ~230 ka, and the 95% confidence interval indicates a time for the onset of growth *no younger *than ~120 kya (or ~30,000 generations). Because we infer such old times for the onset of growth in these bat populations, anthropogenic causes, which began no earlier than 9-15 kya [20,21], are statistically highly unlikely.

Several factors might confound our analysis, although none of these materially affect our main conclusions. First, coalescent analyses assume selective neutrality. There is no evidence for balancing selection (or equivalently, population structure) in our dataset; in such cases, Tajima's *D *trends towards positive values. Positive, directional selection could produce the negative values of Tajima's *D *that we observe, as well as elevate the number of singleton polymorphisms. However, levels of genetic diversity for our loci (e.g., Watterson's theta *θ*_W_, or the number of segregating sites) are not consistent with positive, directional selection, which tends to considerably reduce genetic diversity [28]. Studies in other bat species also fail to reveal consistent evidence of positive selection at these two loci [29-31]. Finally, we note that strong positive, directional selection would lead us to infer high rates of growth and a very recent time of onset - the opposite of what we find here.

Second, both visual inspection of the sequence alignment and tests of the four-gamete rule [32] indicate that the mtDNA locus (and to a lesser extent, *RAG2*) have been affected by homoplasy (or alternately, recombination). Because it recreates polymorphism that is already present in the population, homoplasy tends to reduce the number of segregating sites and singleton polymorphisms that can actually be identified. However, the mutation rates of the two loci studied here - on the order of 10^-8 ^and 10^-10 ^- are sufficiently small that homoplasy should have only minor effects on either dataset, and consequently, on the demographic parameters that we infer.

Third, we may have over- or underestimated generation times. If the average generation interval is actually larger than we assume (i.e., *g *> 4 years), then the time of onset of growth will be proportionally and linearly older than the values we report. Conversely, if the average generation time is less (i.e., *g *< 4 years), then the time of onset of growth will be proportionally and linearly younger. However, even if the average generation time were 1 year, which is not supported by demographic studies in this bat species, our analyses would infer growth beginning no earlier than ~30 kya. This date still long precedes the arrival of humans in the Americas, let alone the emergence of widescale agriculture in North America.

## Conclusions

Our analyses firmly support population growth in Mexican free-tailed bats, but reject a direct co-evolutionary connection with the development of human agriculture. We are then left asking what may have caused this increase in Mexican free-tailed bat numbers. The question is complicated by the fact that our data lack sufficient power to place an upper bound on the time of the onset of growth. However, since we are able to confidently infer a lower bound for this parameter (i.e., growth was no more recent than ~120 ka), we find it likely that the signals of population growth observed in our data may be attributable to range expansion out of Pleistocene refugia. *T. b. mexicana *is a subtropical species, and typically migrates south to overwinter in Mexico (although some populations hibernate in coastal areas; [12]). In glacial periods during the Pleistocene, its range would have been restricted to Central America and Mexico, while interglacial periods would have seen a substantial range expansion and, we expect, concomitant population growth.

Finally, we cannot completely exclude the possibility that growth rates of Mexican free-tailed bat populations may have increased (or indeed decreased) relative to previous levels in response to extremely recent human activity, such as the development of large wind farms or the advent of wide-scale industrialized agriculture following the Second World War. A very recent uptick in the rate of growth on the background of a population that is already growing is extremely difficult to detect [22]. Similarly, it is also difficult to detect very recent decreases in effective population size. With only two genetic loci, we lack sufficient power to detect extremely recent deviations of this nature. Simulation studies indicate that hundreds to thousands of independent loci may be necessary to detect such recent events using sequence data [33]. Identifying very recent changes in population growth is difficult largely because the rate at which such demographic changes are recorded is constrained by low rates of mutation. It remains possible that such recent timescales might be more accessible by studying rapidly evolving microsatellites. Nevertheless, two key points are clear from our analyses: i) Mexican free-tailed bat populations have grown substantially in the past, and ii) this growth began well before humans arrived in the Americas. Given current evidence, it seems most parsimonious to assume that human agricultural activities have not driven this growth process to any major extent.

## Methods

### Samples and sequences

Mexican free-tailed bats (*N_S _*= 94) were sampled from 11 locations throughout Mexico and the southwestern United States (see [23] for locality information). Sample collection protocols were approved by the University of Tennessee Institutional Animal Care and Use Committee (UTK IACUC Protocol #890). Two genetic loci were sequenced in these individuals: a haploid locus, the mitochondrial control region; and a diploid autosomal locus, the Recombination Activating Gene 2 (*RAG2*). We sequenced 474 bp of the mitochondrial DNA (mtDNA) locus in 94 individuals (see methods in [23]), and 686 bp of the *RAG2 *gene (human genome homolog *RAG2*; chr11:36,613,495-36,619,812 in the February 2009 build of the human genome reference, hg19) in 75 individuals (i.e., 150 diploid chromosome copies). The *RAG2 *region was sequenced using primers 179F and 968R [34]. PCR was carried out in 12.5 μL volumes containing 20 ng genomic DNA, 1× PCR Gold buffer (Applied Biosystems), 2.5 mM MgCl_2_, 0.8 mM dNTP Blend (Applied Biosystems) 2.5 ng each primer (Integrated DNA Technologies), and 0.0625 U AmpliTaq Gold DNA polymerase (Applied Biosystems). The PCR amplification profile consisted of initial hot start denaturation for 10 min at 95°C, followed by 35 cycles of denaturation at 95°C for 30 s, annealing at 55°C for 30 s, and elongation at 72°C for 1 min, with a final extension at 72°C for 10 min.

Diploid *RAG2 *sequences were phased into haplotypes using a combination of computational and experimental methods. Fifteen individuals were either homozygous, or heterozygous at a single site, thereby allowing alleles at the *RAG2 *locus to be determined unambiguously. Uncertain diploid sequences were phased computationally using PHASE version 2.1 [35] with five replicate runs of a 2,000 step Markov chain, a burn-in of 1,000 steps, and a thinning interval of 2. We were able to phase 43 individuals computationally using an output probability threshold of 95%. Another 17 individuals, for which haplotypes were more ambiguous when subjected to computational phasing, were phased experimentally using standard cloning techniques. We first incubated 5 μL of PCR product with 1 U GoTaq Flexi polymerase (Promega) at 72°C for 10 min to add 3' dATPs for the TA cloning protocol. One μL of this reaction product was used for TOPO TA cloning (Invitrogen), from which plasmids were harvested from 3-12 *E. coli *colonies using the FastPlasmid Mini Purification System (5 Prime). Plasmids were sequenced using the BigDye v. 3.1 Terminator Cycle Sequencing Kit (Applied Biosystems) and the M13R primer (5'-CAGGAAACAGCTATGAC-3') on an ABI 3100 automated sequencer (Applied Biosystems). All sequences were edited and aligned using Sequencher v. 4.5 (GeneCodes). Sequence data are available online (GenBank: AY348008-AY348101, HM592833-HM592982).

### Summary statistics

A suite of summary statistics was assembled to describe patterns of variation at each locus. Watterson's theta *θ_W_*, which is calculated from the number of segregating sites *S*, summarizes the total length of the genealogy [36], while the average number of pairwise differences *θ_π _*summarizes the average coalescent time [37]. Both summary statistics are unbiased moment estimators of the population mutation rate (*θ *= *sN_e_μ*), where *s *is the genomic scalar (*s *= 1 for uniparentally-inherited haploid loci, *s *= 4 for biparentally-inherited autosomal loci), *N_e _*is the effective population size, and *μ *is the mutation rate. Tajima's *D *[38] is the normalized difference between these two estimators, *θ_π_*-*θ_W_*, and has an expectation of zero under the standard neutral model. Non-zero values indicate deviations from this model. One such deviation is population growth, which tends to increase the number of singletons *η_1 _*present in a sample (i.e., derived polymorphisms identified in only one individual). Because singletons elevate *θ_W _*relative to *θ_π_*, growing populations are often characterized by negative values of Tajima's *D*. Similarly, an increased number of singletons also tends to elevate the number of unique haplotypes *h*. Therefore, all of these summaries were calculated for observed and simulated data using the C++ library *libsequence *[39].

The *RAG2 *mutation rate of 8.6 × 10^-10 ^substitutions/site/year was estimated by comparing data for *T*. *b. mexicana *with an outgroup sequence from *Eumops auripendulus *(GenBank: AY834668) and assuming a divergence time of 22 mya [40]. The control region mutation rate was taken from published estimates for *T. b. mexicana *(2.0 × 10^-8 ^substitutions/site/year; [23]). Although the generation time of *T*. *b. mexicana *is not known with certainty, individuals reach sexual maturity at ~1 year, and have been known to live for at least 8 years in nature [41]. In our analyses, we initially assume a mean generation interval of 4 years (specifically, the average age of parents across all offspring, rather than the age at which parents first reproduce). The recombination rate for *RAG2 *was inferred directly from the autosomal sequence data using *PHASE *[42].

### Population growth model

To provide more nuanced inference than is possible from simple observation of summary statistics, we compared the data to a two-phase population growth model (Figure 1). Under this model, populations experience an early period of constant size (Phase 1), followed by a period of exponential growth (Phase 2). This model has three parameters: the ancestral population size *N_A_*, the modern population size *N_0_*, and the time of onset of growth *τ*. Hence, considered in a backwards-in-time framework, the population size at any time *t *is provided by(1)

where *α *is the exponential growth rate. Note that the scenario of constant effective size (i.e., *N_0 _*= *N_A_*) is nested within this growth model (i.e., *α *= 0). Previous analyses of this subspecies are consistent with panmixia [23,43], so we do not consider the effects of population structure in our model.

### Demographic inference

To infer demographic parameters under the two-phase population growth model, we employed an inference procedure based on the Maximum Likelihood framework. Likelihood functions for complex demographic histories are too intractable to be derived analytically, and we therefore determine likelihoods by simulation with the *n*-coalescent under the infinite sites model [44]. Our approach thus belongs to a class of *approximate *methods [27]. Likelihoods are estimated from three summary statistics calculated from the data: the number of segregating sites *S*, which controls for the population mutation rate *θ*; and the number of singletons *η_1 _*and haplotypes *h*, both of which vary proportionally with population growth. All three summary statistics have the added convenience that they increase in integer units (i.e., *S*, *η_1_*, *h *∈ **ℕ**), which significantly raises the number of instances where the values of these summary statistics are identical in both observed and simulated datasets (see below). This characteristic greatly simplifies the calculation of likelihoods.

To explain the method simply, we aim to estimate the likelihood of a set of observed summary statistics *ϕ *= {*S*, *η*_1, _*h*} across the parameter space of the two-phase growth model Λ = {*N*_*A*_, *N*_0_, *τ*}. If the likelihood surface is known, it is a relatively trivial matter to determine the set of demographic parameters  that maximizes the likelihood of the observed set of summary statistics *ϕ*. (This is the maximum likelihood estimate, or MLE, of *N_A_*, *N_0 _*and *τ*). In practice, however, determining the complete likelihood surface is both difficult and computationally expensive. To generate an approximate alternative, we set initial bounds on the parameter space *Λ *such that *N_A _*∈ {10^4^, 10^6^}, *N_0 _*∈ {10^4^, 5×10^7^}, and *τ *∈ {0, 5×10^5^}. We produced a uniform 10 × 10 × 10 grid across this 3-dimensional parameter space, although to ensure coalescence, we constrained the parameter space such that *N_0 _*≥ *N_A _*(or equivalently, *α *≥ 0). In comparison, *τ *was allowed to vary freely across its full range. At each point in the grid, we sampled 10^6 ^coalescent trees for the haploid locus and 10^6 ^ancestral recombination graphs (ARGs) for the autosomal locus using the simulation software *ms *[45]. These simulations were conditioned on observed sequence lengths, inferred mutation rates, and for the autosomal locus, the inferred recombination rate. Instances matching the observed values of *S*, *η_1_*, and *h *(i.e., the likelihoods of *S*, *η_1 _*and *h*) were counted directly from these simulated distributions. A combined likelihood surface was then constructed by multiplying likelihoods at each grid point across all loci *i *and all summary statistics *j*(2)

where *f_λ _*is the observed marginal coalescent likelihood of *ϕ_ij_*. The MLE is simply the set of demographic parameters  that maximizes *L*(*λ*). Although calculating the joint coalescent likelihood of *ϕ_ij _*would be preferable, this would require orders of magnitude more computational time than the method applied here and is not currently computationally feasible. Validation simulations (see below) show that using marginal values does not affect the accuracy of our method.

As is traditional, we report the natural log of likelihoods in preference to the likelihoods themselves; that is, values are reported on the scale (-∞, 0) rather than (0, 1). Confidence intervals were constructed using standard methods. In brief, confidence intervals incorporate all values *x *such that(3)

Because we consider a parameter space with three independent demographic parameters, the quartile of the χ^2 ^distribution with 3 degrees of freedom and a one-tailed probability of 0.05 was applied. Profile likelihoods were also calculated using standard methods; however, because these confidence intervals represent only a single dimension (i.e., each demographic parameter is considered separately), we applied a χ^2 ^distribution with only 1 degree of freedom.

### Validation

Our inference method was validated by generating 10^3 ^coalescent trees and ARGs using values of *N_A_*, *N_0 _*and *τ *chosen randomly from a uniform distribution across the parameter space. We applied the approximate Maximum Likelihood method described above to the values of *S*, *η_1 _*and *h *returned by each simulation. Instances where the known (i.e., simulated) values of *N_A_*, *N_0 _*and *τ *were included within the confidence intervals of the inferred demography were considered successful. Deviation from expectation is reported as a type I error rate.

## Authors' contributions

ALR, MPC, and GFM conceived of the study and participated in its design. ALR and VAB carried out the molecular genetic studies. ALR aligned the sequence data and performed initial statistical analyses. MPC wrote code for and performed the approximate Maximum Likelihood analyses. ALR and MPC drafted the manuscript. All authors read and approved the final manuscript.

## Supplementary Material

Additional file 1 **Log-likelihood surface (*N_A _*versus τ) for the haploid mtDNA control region**. Click here for file

Additional file 2 **Log-likelihood surface (*N_A _*versus τ) for the autosomal *RAG2 *locus**. Click here for file

Additional file 3 **Grid points forming the 95% confidence interval of the three-dimensional parameter space ranked by likelihood value**. Click here for file

Additional file 4 **Profile likelihood curves drawn from the combined likelihood surface for the haploid mtDNA control region and autosomal *RAG2 *locus**. Click here for file

## References

[B1] Currey RJC Dawson SM Slooten E Schneider K Lusseau D Boisseau OJ Haase P Williams JA Survival rates for a declining population of bottlenose dolphins in Doubtful Sound, New Zealand: an information theoretic approach to assessing the role of human impacts Aquat Conserv 2009 19 658 670 10.1002/aqc.1015

[B2] Stallings CD Fishery-independent data reveal negative effect of human population density on Caribbean predatory fish communities PLoS One 2009 4 e5333 10.1371/journal.pone.0005333 19421312 PMC2672166

[B3] Regular PM Robertson GJ Montevecchi WA Shuhood F Power T Ballam D Piatt JF Relative importance of human activities and climate driving common murre population trends in the Northwest Atlantic Polar Biol 2010 33 1215 1226 10.1007/s00300-010-0811-2

[B4] May GE Gelembiuk GW Panov VE Orlova MI Lee CE Molecular ecology of zebra mussel invasions Mol Ecol 2006 15 1021 1031 10.1111/j.1365-294X.2006.02814.x 16599964

[B5] Hulva P Fornusková A Chudárková A Evin A Allegrini B Benda P Bryja J Mechanisms of radiation in a bat group from the genus *Pipistrellus *inferred by phylogeography, demography and population genetics Mol Ecol 2010 19 5417 5431 10.1111/j.1365-294X.2010.04899.x 21054608

[B6] Purcell JE Uye S Lo WT Anthropogenic causes of jellyfish blooms and their direct consequences for humans: a review Mar Ecol Prog Ser 2007 350 153 174 10.3354/meps07093

[B7] Hugo S van Rensburg BJ The maintenance of a positive spatial correlation between South African bird species richness and human population density Global Ecol Biogeogr 2008 17 611 621 10.1111/j.1466-8238.2008.00391.x

[B8] Goossens B Chikhi L Ancrenaz M Lackman-Ancrenaz I Andau P Bruford MW Genetic signature of anthropogenic population collapse in orang-utans PLoS Biol 2006 4 e25 10.1371/journal.pbio.0040025 16417405 PMC1334199

[B9] Liu Z Ren B Wu R Zhao L Hao Y Wang B Wei F Long Y Li M The effect of landscape features on population genetic structure in Yunnan snub-nosed monkeys (*Rhinopithecus bieti*) implies an anthropogenic genetic discontinuity Mol Ecol 2009 18 3831 3846 10.1111/j.1365-294X.2009.04330.x 19732331

[B10] Funk WC Forsman ED Johnson M Mullins TD Haig SM Evidence for recent population bottlenecks in northern spotted owls (*Strix occidentalis caurina*) Conserv Genet 2010 11 1013 1021 10.1007/s10592-009-9946-5

[B11] Davis RB Herreid CF Short HL Mexican free-tailed bats in Texas Ecol Monogr 1962 32 311 346 10.2307/1942378

[B12] Cockrum EL Migration in the guano bat, *Tadarida brasiliensis* Misc Pub Univ Kansas Mus Nat Hist 1969 51 303 336

[B13] Betke M Hirsh DE Makris NC McCracken GF Procopio M Hristov NI Tang S Bagchi A Reichard JD Horn JW Crampton S Cleveland CJ Kunz TH Thermal imaging reveals significantly smaller Brazilian free-tailed bat colonies than previously estimated J Mammal 2008 89 18 24 10.1644/07-MAMM-A-011.1

[B14] Kunz TH Whitaker JO Wadanoli MD Dietary energetics of the insectivorous Mexican free-tailed bat (*Tadarida brasiliensis*) during pregnancy and lactation Oecologia 1995 101 407 415 10.1007/BF00329419 28306955

[B15] Gatehouse AG Behavior and ecological genetics of wind-borne migration by insects Annu Rev Entomol 1997 42 475 502 10.1146/annurev.ento.42.1.475 15012321

[B16] McCracken GF Gillam EH Westbrook JK Lee YF Jensen ML Balsley BB Brazilian free-tailed bats (*Tadarida brasiliensis*: Molossidae, Chiroptera) at high altitude: links to migratory insect populations Integr Comp Biol 2008 48 107 118 10.1093/icb/icn033 21669777

[B17] McCracken GF Brown VA Eldridge M Federico P Westbrook JK Opportunistic predation by bats tracks and exploits changes in insect pest populations: evidence from quantitative (qPCR) analysis of fecal DNA Bat Res News 2008 49 147

[B18] Lee YF McCracken GF Dietary variation of Brazilian free-tailed bats links to migratory populations of pest insects J Mammal 2005 86 67 76 10.1644/1545-1542(2005)086<0067:DVOBFB>2.0.CO;2

[B19] Cleveland CJ Betke M Federico P Frank JD Hallam TG Horn J López JD McCracken GF Medellín RA Moreno-Valdez A Sansone CG Westbrook JK Kunz TH Economic value of the pest control service provided by Brazilian free-tailed bats in south-central Texas Front Ecol Environ 2006 4 238 243 10.1890/1540-9295(2006)004[0238:EVOTPC]2.0.CO;2

[B20] Piperno DR Ranere AJ Holst I Iriarte J Dickau R Starch grain and phytolith evidence for early ninth millennium B.P. maize from the Central Balsas River Valley, Mexico Proc Natl Acad Sci USA 2009 106 5019 5024 10.1073/pnas.0812525106 19307570 PMC2664021

[B21] Gilbert MTP Jenkins DL Götherström A Naveran N Sanchez JJ Hofreiter M Thomsen PF Binladen J Higham TFG Yohe RM Parr R Cummings LS Willerslev E DNA from pre-Clovis human coprolites in Oregon, North America Science 2008 320 786 789 10.1126/science.1154116 18388261

[B22] Cox MP Morales DA Woerner AE Sozanski J Wall JD Hammer MF Autosomal resequence data reveal late Stone Age signals of population expansion in sub-Saharan African foraging and farming populations PLoS One 2009 4 e6366 10.1371/journal.pone.0006366 19641603 PMC2712685

[B23] Russell AL Medellín RA McCracken GF Genetic variation and migration in the Mexican free-tailed bat (*Tadarida brasiliensis mexicana*) Mol Ecol 2005 14 2207 2222 10.1111/j.1365-294X.2005.02552.x 15910338

[B24] Metni Pilkington M Wilder JA Mendez FL Cox MP Woerner A Angui T Kingan S Mobasher Z Batini C Destro-Bisol G Soodyall H Strassmann BI Hammer MF Contrasting signatures of population growth for mitochondrial DNA and Y chromosomes among human populations in Africa Mol Biol Evol 2008 25 517 525 10.1093/molbev/msm279 18093995

[B25] Pluzhnikov A Di Rienzo A Hudson RR Inferences about human demography based on multilocus analyses of noncoding sequences Genetics 2002 161 1209 1218 12136023 10.1093/genetics/161.3.1209 PMC1462170

[B26] Voight BF Adams AM Frisse LA Qian Y Hudson RR Di Rienzo A Interrogating multiple aspects of variation in a full resequencing data set to infer human population size changes Proc Natl Acad Sci USA 2005 102 18508 18513 10.1073/pnas.0507325102 16352722 PMC1311907

[B27] Plagnol V Wall JD Possible ancestral structure in human populations PLoS Genet 2006 2 e105 10.1371/journal.pgen.0020105 16895447 PMC1523253

[B28] Peng Y Shi H Qi XB Xiao CJ Zhong H Ma RL Su B The ADH1B Arg47His polymorphism in East Asian populations and expansion of rice domestication in history BMC Evol Biol 2010 10 15 10.1186/1471-2148-10-15 20089146 PMC2823730

[B29] Lamb JM Ralph TMC Goodman SM Bogdanowicz W Fahr J Gajewska M Bates PJJ Eger J Benda P Taylor PJ Phylogeography and predicted distribution of African-Arabian and Malagasy populations of giant mastiff bats, *Otomops *spp. (Chiroptera: Molossidae) Acta Chiropterol 2008 10 21 40 10.3161/150811008X331063

[B30] Juste J Bilgin R Muñoz J Ibáñez C Mitochondrial DNA signatures at different spatial scales: from the effects of the Straits of Gibraltar to population structure in the meridional serotine bat (*Eptesicus isabellinus*) Heredity 2009 103 178 187 10.1038/hdy.2009.47 19401715

[B31] Martins FM Templeton AR Pavan ACO Kohlbach BC Morgante JS Phylogeography of the common vampire bat (*Desmodus rotundus*): marked population structure, Neotropical Pleistocene vicariance and incongruence between nuclear and mtDNA markers BMC Evol Biol 2010 9 294 10.1186/1471-2148-9-294 PMC2801518 20021693

[B32] Woerner AE Cox MP Hammer MF Recombination-filtered genomic datasets by information maximization Bioinformatics 2007 23 1851 1853 10.1093/bioinformatics/btm253 17519249

[B33] Adams AM Hudson RR Maximum-likelihood estimation of demographic parameters using the frequency spectrum of unlinked single-nucleotide polymorphisms Genetics 2004 168 1699 1712 10.1534/genetics.104.030171 15579718 PMC1448761

[B34] Stadelmann B Lin LK Kunz TH Ruedi M Molecular phylogeny of New World *Myotis *(Chiroptera, Vespertilionidae) inferred from mitochondrial and nuclear DNA genes Mol Phylogenet Evol 2007 43 32 48 10.1016/j.ympev.2006.06.019 17049280

[B35] Stephens M Smith NJ Donnelly P A new statistical method for haplotype reconstruction from population data Am J Hum Genet 2001 68 978 989 10.1086/319501 11254454 PMC1275651

[B36] Watterson GA On the number of segregating sites in genetical models without recombination Theor Popul Biol 1975 7 256 276 10.1016/0040-5809(75)90020-9 1145509

[B37] Tajima F Evolutionary relationship of DNA sequences in finite populations Genetics 1983 105 437 460 6628982 10.1093/genetics/105.2.437 PMC1202167

[B38] Tajima F Statistical method for testing the neutral mutation hypothesis by DNA polymorphism Genetics 1989 123 585 595 2513255 10.1093/genetics/123.3.585 PMC1203831

[B39] Thornton K libsequence: a C++ class library for evolutionary genetic analysis Bioinformatics 2003 19 2325 2327 10.1093/bioinformatics/btg316 14630667

[B40] Teeling EC Springer MS Madsen O Bates P O'Brien SJ Murphy WJ A molecular phylogeny for bats illuminates biogeography and the fossil record Science 2005 307 580 584 10.1126/science.1105113 15681385

[B41] Brunet-Rossinni A Austad S Ageing studies on bats: a review Biogerontol 2004 5 211 222 10.1023/B:BGEN.0000038022.65024.d8 15314271

[B42] Li N Stephens M Modelling linkage disequilibrium, and identifying recombination hotspots using SNP data Genetics 2003 165 2213 2233 14704198 10.1093/genetics/165.4.2213 PMC1462870

[B43] McCracken GF McCracken MK Vawter AT Genetic structure in migratory populations of the bat *Tadarida brasiliensis mexicana* J Mammal 1994 75 500 514 10.2307/1382574

[B44] Wakeley J Coalescent Theory: An Introduction 2008 Greenwood Village: Roberts & Company Publishers

[B45] Hudson RR Generating samples under a Wright-Fisher neutral model of genetic variation Bioinformatics 2002 18 337 338 10.1093/bioinformatics/18.2.337 11847089

