# Supplementary material for: Population growth of Mexican free-tailed bats (Tadarida brasiliensis mexicana) predates human agricultural activity
Source: BMC Evol Biol. 2011 Apr 1;11:88. doi: 10.1186/1471-2148-11-88 (PMC3080819; doi:10.1186/1471-2148-11-88)
Supplement: Additional file 1 — Log-likelihood surface (NA versus τ) for the haploid mtDNA control region. [file 1471-2148-11-88-S1.PDF]

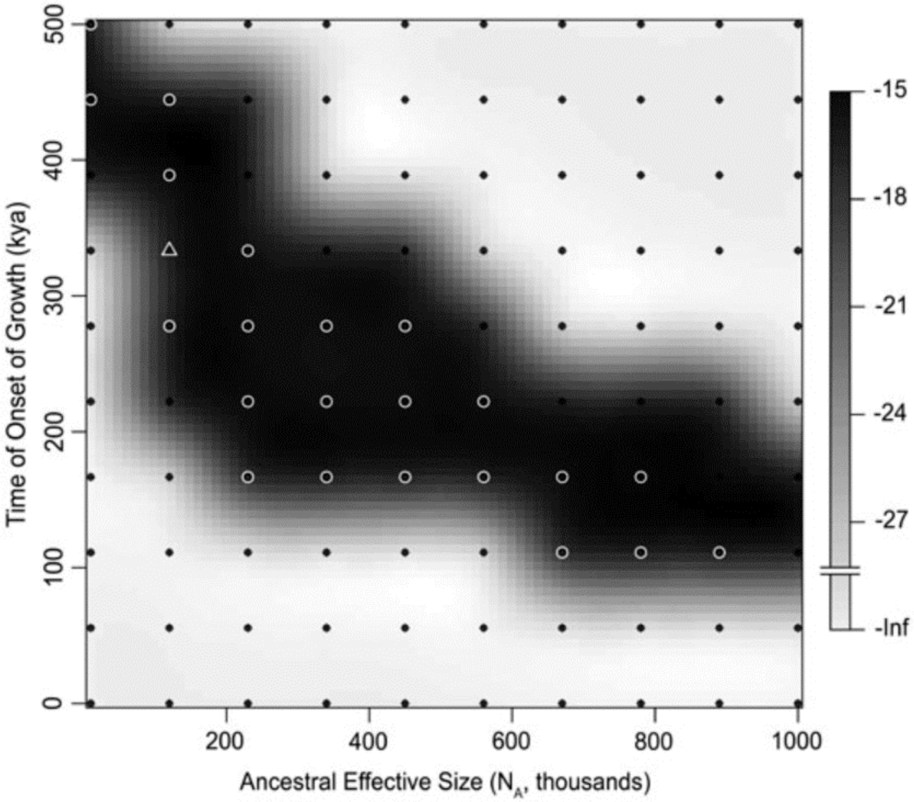

Additional file 1. Log-likelihood surface (N_A versus τ) for the haploid mtDNA control region. Black and white points indicate the grid of sampling locations. Log-likelihoods at these points are known with certainty, whereas log-likelihoods in the intervening space are interpolated. Regions of the parameter space with highest likelihood are shaded black. Only highlighted white points (circles and triangles) fall within the 95% confidence interval. The maximum likelihood estimate (MLE) is indicated by a white triangle. N_0 was set to its value for the MLE.
