# Supplementary material for: Population growth of Mexican free-tailed bats (Tadarida brasiliensis mexicana) predates human agricultural activity
Source: BMC Evol Biol. 2011 Apr 1;11:88. doi: 10.1186/1471-2148-11-88 (PMC3080819; doi:10.1186/1471-2148-11-88)
Supplement: Additional file 3 — Grid points forming the 95% confidence interval of the three-dimensional parameter space ranked by likelihood value. [file 1471-2148-11-88-S3.PDF]

Additional file 3. Grid points forming the 95% Confidence Interval of the three-dimensional parameter space ranked by likelihood value. The Maximum Likelihood estimate (MLE) is shown in bolded italics, and points within the 95% confidence area that are plotted in Fig. 2 are highlighted.

N_A	N_0	τ (years)	$L^*(\lambda)$
230,000	***11,118,889***	***222,223***	***-27.06***
230,000	16,673,333	222,223	-27.13
340,000	22,227,778	166,667	-27.34
230,000	44,445,556	166,667	-27.40
230,000	22,227,778	166,667	-27.48
230,000	27,782,222	166,667	-27.52
230,000	33,336,667	166,667	-27.53
340,000	50,000,000	111,112	-27.54
230,000	38,891,111	166,667	-27.55
230,000	11,118,889	277,778	-27.57
340,000	16,673,333	166,667	-27.71
340,000	27,782,222	166,667	-27.74
340,000	33,336,667	166,667	-27.82
230,000	5,564,444	333,334	-27.82
230,000	27,782,222	222,223	-27.91
230,000	50,000,000	166,667	-27.92
120,000	11,118,889	333,334	-28.05
340,000	11,118,889	222,223	-28.10
340,000	38,891,111	111,112	-28.13
340,000	38,891,111	166,667	-28.26
340,000	11,118,889	166,667	-28.28
230,000	16,673,333	166,667	-28.30
230,000	22,227,778	222,223	-28.32
340,000	44,445,556	166,667	-28.37
340,000	5,564,444	277,778	-28.54
120,000	5,564,444	500,000	-28.62
230,000	5,564,444	277,778	-28.65
340,000	44,445,556	111,112	-28.70
230,000	5,564,444	388,889	-28.78
120,000	16,673,333	277,778	-28.86
230,000	11,118,889	166,667	-29.06
340,000	33,336,667	111,112	-29.08
120,000	5,564,444	444,445	-29.11
120,000	11,118,889	388,889	-29.15
450,000	50,000,000	111,112	-29.16
230,000	11,118,889	333,334	-29.21
120,000	11,118,889	277,778	-29.24
340,000	16,673,333	222,223	-29.25
120,000	22,227,778	277,778	-29.35
340,000	50,000,000	166,667	-29.45
230,000	16,673,333	277,778	-29.62
450,000	44,445,556	111,112	-29.67
450,000	38,891,111	111,112	-29.71
230,000	5,564,444	444,445	-29.83
450,000	27,782,222	111,112	-29.86
450,000	33,336,667	111,112	-29.95
120,000	22,227,778	222,223	-30.03
120,000	27,782,222	222,223	-30.04
120,000	5,564,444	388,889	-30.19
340,000	22,227,778	222,223	-30.22
120,000	5,564,444	333,334	-30.23
120,000	38,891,111	222,223	-30.26
120,000	33,336,667	277,778	-30.30
450,000	11,118,889	166,667	-30.31
340,000	5,564,444	333,334	-30.35
450,000	16,673,333	166,667	-30.47
120,000	16,673,333	222,223	-30.48
120,000	33,336,667	222,223	-30.55
340,000	11,118,889	277,778	-30.62
450,000	22,227,778	111,112	-30.63
230,000	5,564,444	500,000	-30.90
340,000	27,782,222	222,223	-30.91
450,000	22,227,778	166,667	-30.93
