# Supplementary material for: Population growth of Mexican free-tailed bats (Tadarida brasiliensis mexicana) predates human agricultural activity
Source: BMC Evol Biol. 2011 Apr 1;11:88. doi: 10.1186/1471-2148-11-88 (PMC3080819; doi:10.1186/1471-2148-11-88)
Supplement: Additional file 4 — Profile likelihood curves drawn from the combined likelihood surface for the haploid mtDNA control region and autosomal RAG2 locus. [file 1471-2148-11-88-S4.PDF]

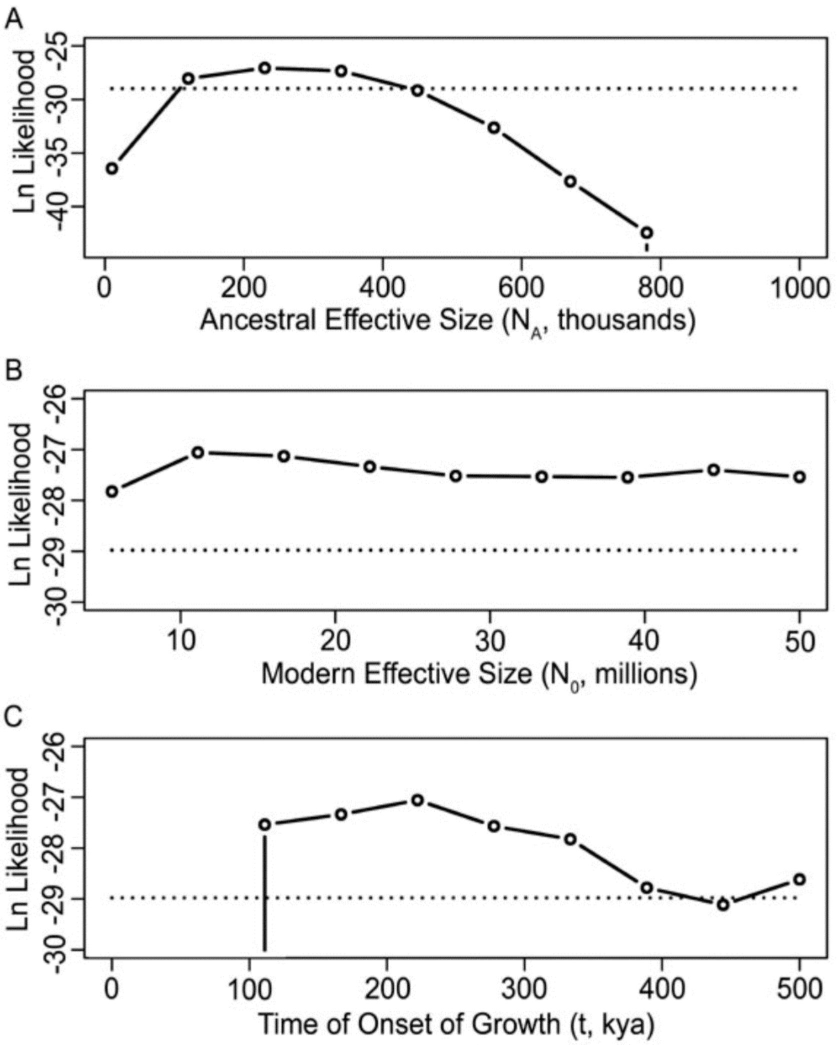

Additional file 4. Profile likelihood curves drawn from the combined likelihood surface for the haploid mtDNA control region and autosomal *RAG2* locus. Dotted lines indicate the 95% confidence interval. Note that there is little power to infer modern effective sizes, and that an onset of growth for all times less than ~100 kya is statistically unlikely.
